# Rosemary extract improves egg quality by altering gut barrier function, intestinal microbiota and oviductal gene expressions in late-phase laying hens

**DOI:** 10.1186/s40104-023-00904-6

**Published:** 2023-09-04

**Authors:** Lianhua Zhang, Junwei Ge, Fei Gao, Min Yang, Hui Li, Fei Xia, Hongtong Bai, Xiangshu Piao, Zhiying Sun, Lei Shi

**Affiliations:** 1 Key Laboratory of Plant Resources, Institute of Botany, Chinese Academy of Sciences, Beijing, 100093 China; 2 China National Botanical Garden, Beijing, 100093 China; 3 College of Pharmacy, Shandong University of Traditional Chinese Medicine, Jinan, 250355 China; 4 University of Chinese Academy of Sciences, Beijing, 100049 China; 5 State Key Laboratory of Animal Nutrition, College of Animal Science and Technology, China Agricultural University, Beijing, 100193 China

**Keywords:** Intestinal health, Laying hens, Microbiota, Oviductal function, Phytochemicals, Production

## Abstract

**Background:**

Rosemary extract (RE) has been reported to exert antioxidant property. However, the application of RE in late-phase laying hens on egg quality, intestinal barrier and microbiota, and oviductal function has not been systematically studied. This study was investigated to detect the potential effects of RE on performance, egg quality, serum parameters, intestinal heath, cecal microbiota and metabolism, and oviductal gene expressions in late-phase laying hens. A total of 210 65-week-old “Jing Tint 6” laying hens were randomly allocated into five treatments with six replicates and seven birds per replicate and fed basal diet (CON) or basal diet supplemented with chlortetracycline at 50 mg/kg (CTC) or RE at 50 mg/kg (RE50), 100 mg/kg (RE100), and 200 mg/kg (RE200).

**Results:**

Our results showed that RE200 improved (*P* < 0.05) Haugh unit and n-6/n-3 of egg yolk, serum superoxide dismutase (SOD) compared with CON. No significant differences were observed for Haugh unit and n-6/n-3 of egg yolk among CTC, RE50, RE100 and RE200 groups. Compared with CTC and RE50 groups, RE200 increased serum SOD activity on d 28 and 56. Compared with CON, RE supplementation decreased (*P* < 0.05) total cholesterol (TC) level. CTC, RE100 and RE200 decreased (*P* < 0.05) serum interleukin-6 (IL-6) content compared with CON. CTC and RE200 increased jejunal mRNA expression of *ZO-1* and *Occludin* compared with CON. The biomarkers of cecal microbiota and metabolite induced by RE 200, including Firmicutes, *Eisenbergiella*, *Paraprevotella*, *Papillibacter*, and butyrate, were closely associated with Haugh unit, n-6/n-3, SOD, IL-6, and TC. PICRUSt2 analysis indicated that RE altered carbohydrate and amino acid metabolism of cecal microbiota and increased butyrate synthesizing enzymes, including 3-oxoacid CoA-transferase and butyrate-acetoacetate CoA-transferase. Moreover, transcriptomic analysis revealed that RE200 improved gene expressions and functional pathways related to immunity and albumen formation in the oviductal magnum.

**Conclusions:**

Dietary supplementation with 200 mg/kg RE could increase egg quality of late-phase laying hens via modulating intestinal barrier, cecal microbiota and metabolism, and oviductal function. Overall, RE could be used as a promising feed additive to improve egg quality of laying hens at late stage of production.

**Graphical Abstract:**

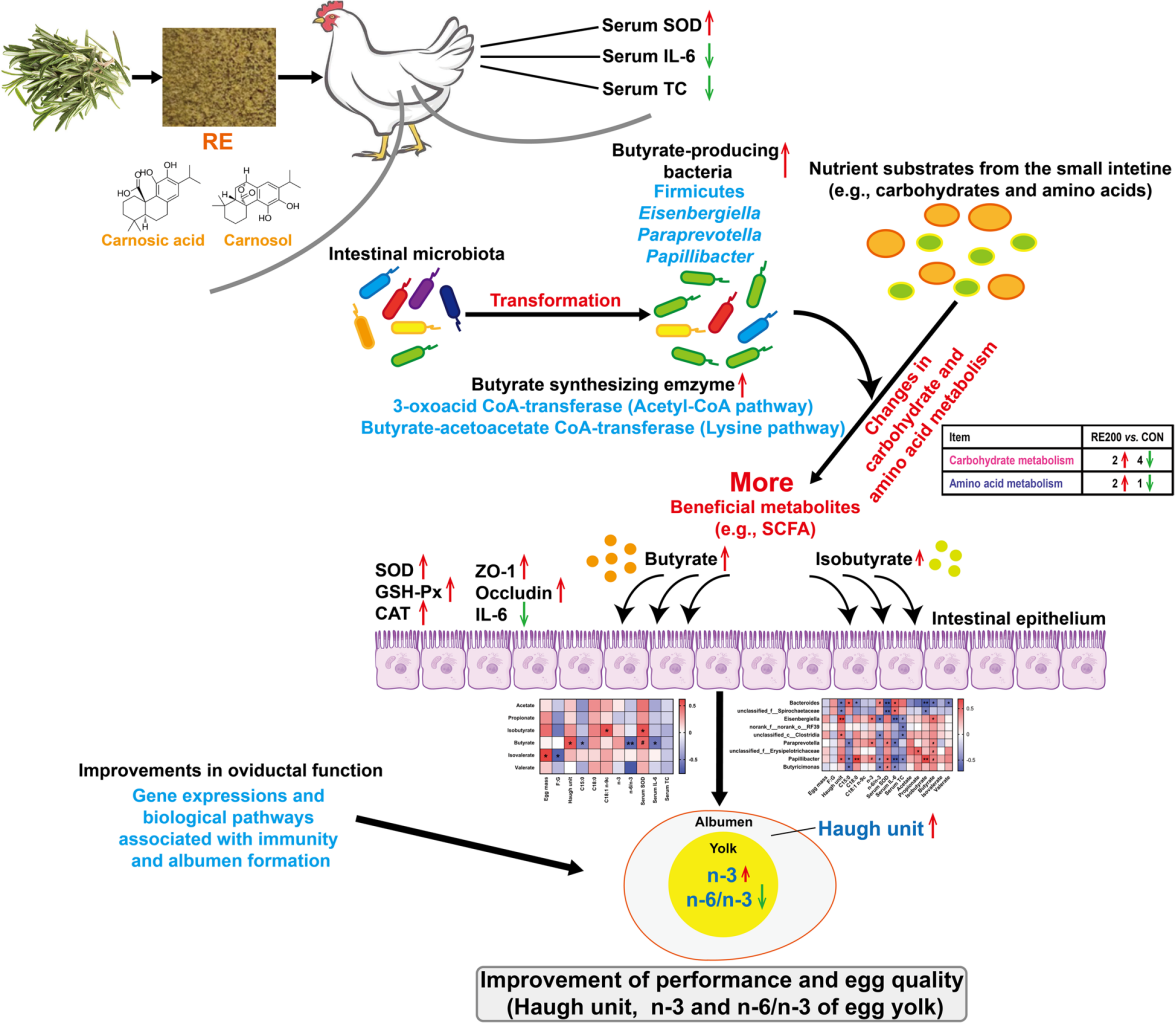

**Supplementary Information:**

The online version contains supplementary material available at 10.1186/s40104-023-00904-6.

## Introduction

Eggs are an important food source, which can provide human with high-quality protein and sufficient vitamins and mineral elements. Late-phase laying hens usually face various stressors, such as excessive accumulation of reactive oxygen species (ROS), imbalance of redox process, weakening of reproductive system function, resulting in egg albumen dilution and impaired Haugh unit and albumen height [1]. In addition, late stage of production can lead to intestinal damage, immune imbalance and gut microbiota disturbance of laying hens [2]. Owing to the restriction of antibiotic growth promoters for animals, it is critical to explore natural bioactive compounds for improving poultry production.

Rosemary (*Rosmarinus officinalis* L.) is a medicinal plant in the Lamiaceae family, which is native to the Mediterranean and widely cultivated throughout the world. Rosemary extract (RE) contains non-volatile active ingredients, which have various pharmacological properties such as antioxidant [3], anti-inflammatory [4], antibacterial [5], anti-apoptotic [6], and anti-cancer effects [7]. The bioactive components of RE are mainly diterpenoid phenols, including carnosic acid, carnosol, rosmanol, epirosmanol, rosmaridiphenol [8], and phenolic acids, including rosmarinic acid, ferulic acid, chlorogenic acid, and caffeic acid [9]. Given this, rosemary has been widely used in food preservation and pharmaceutical industry because rosemary is a common, available, and non-toxic herb. Several studies demonstrated that RE modulated intestinal microbiota and improved the performance, antioxidant status, and immunity of mice [10], weaning pigs [11], and broiler chickens [12], indicating that RE showed great potential as natural feed additives in the animal husbandry. Dietary RE supplementation at variable concentrations could improve feed conversion ratio, increased serum levels of total antioxidant capacity (T-AOC), catalase (CAT), glutathione peroxidase (GSH-Px), superoxide dismutase (SOD), immunoglobulin A (IgA), IgG, and IgM, and reduced the relative abundances of specific bacteria negatively correlated to antioxidative and immune-associated parameters, such as *Lachnoclostridium*, *Escherichia_Shigella*, and *Marvinbryantia* in the cecum of broilers [12]. However, little information is available regarding the effects of RE at variable concentrations used in late-phase laying hens on laying performance, egg quality, intestinal microbiota, and oviductal function. In the present study, we hypothesized that dietary supplementation with RE would positively promote gut barrier function, intestinal microbiota, and oviductal gene expressions, subsequently contributing to the improvements of egg quality in late-phase laying hens. Therefore, this research was investigated to study the effects of RE on production performance, selected egg quality traits, serum parameters, jejunal mRNA expression of immune-related and tight junction-related genes, intestinal microbiota, and transcriptomic profiling of oviductal magnum in late-phase laying hens and to provide a theoretical basis for the application of RE in laying hens.

## Materials and methods

### Preparation of RE

The plant material of rosemary used in the study was obtained from Fuyang Base of National Aromatic Plant Germplasm Resource Bank (Anhui, China). The dry powder of rosemary leaves was mixed with 95% ethanol at a ratio of 1:4 (w/v), and extracted by rotary stirring for 90 min. The extraction was repeated three times and decolorized with activated carbon. After evaporated under specific pressure (32 kPa) at 60 °C with a rotary evaporator, RE was obtained by freeze-drying process. As demonstrated by Liu et al. [13], RE was prepared into methanol solution with concentration of 10 mg/mL, filtered by 0.22-μm filter membrane, and then added into injection vial. The absorption peak areas of carnosic acid and carnol were determined by high performance liquid chromatography (UtiMate 3000, Thermo Fisher, Waltham, MA, USA) with a C18 column (4.60 mm × 250 mm, 5 μm). The mobile phase A consisted of water containing 0.1% phosphoric acid and mobile phase B consisted of acetonitrile containing 0.1% phosphoric acid (60:40). The contents of carnosic acid and carnol in the sample were calculated according to the standard curve. The RE product contained 13.27% carnosic acid and 13.20% carnol.

### Animals and experimental treatment

This study was granted by the Institutional Animal Care and Use Committee of China Agricultural University (Beijing, China; No. AW42601202-1-1). A total of 210 healthy 65-week-old “Jing Tint 6” laying hens were randomly allocated into 5 treatments with 6 replicates and 7 birds per replicate: (1) CON, control group; (2) CTC, 50 mg/kg chlortetracycline; (3) RE50, 50 mg/kg rosemary extract; (4) RE100, 100 mg/kg rosemary extract; (5) RE200, 200 mg/kg rosemary extract. The adaptation period lasted one week and the formal experiment lasted 8 weeks. Before conducting the formal experiment, egg production and quality was assessed so that there was no statistical difference among all treatments. All birds were obtained from Gu’an Songhe Poultry Breeding Co., Ltd. (Hebei, China) and reared up in wire-floored cages (0.9 m × 0.6 m × 0.4 m) and allowed mash feed and water ad libitum with exposure to 16 h of light/d. The temperature was controlled at approximately 23 °C, and the birds were immunized according to the routine immunization procedure. The feed formulation is present in Table S1 based on the Chinese Feeding Standard of Chicken (NY/T33-2004) [14]. In this study, feed intake was determined weekly, and egg weight and production were recorded daily.

### Sample collection

On d 28 and 56, one bird per pen (6 birds per treatment) was selected. The blood samples were taken from the wing veins and serum was obtained after centrifugation (5810, Eppendorf Corporate, Hamburg, Germany) at 3,000 × *g* for 10 min to isolate. On d 56, three eggs of each pen were used to determine egg quality, and 2 eggs were randomly gathered from each pen to separate egg yolk. Birds (one bird from each replicate) were slaughtered by cervical dislocation. The jejunal mucosa was scratched gently from the middle part and stored at −80 °C. The cecal digesta and magnum tissues were harvested for analyzing gut microbiota and transcriptomic profiling.

### Egg quality and fatty acids of egg yolk

Yolk color, Haugh unit, and albumen height were detected using an Egg Analyzer (EA-01, Israel Orka Food Technology Ltd., Bountiful, UT, USA). Eggshell thickness and strength were detected by Egg Shell Thickness Gauge (ESTG-1, Israel Orka Food Technology Ltd., Bountiful, UT, USA) and Egg Force Reader (EFR-01, Israel Orka Food Technology Ltd., Bountiful, UT, USA), respectively. The fatty acid profile was determined as reported by Zhang et al. [15]. Briefly, lyophilized egg yolk (200 mg) was mixed with 1 mL n-hexane, 4 mL methanolic HCl solution, and 1 mL internal standard [1 mg/mL 11 carbon fatty acid methyl ester (FAME)], and then the mixture was maintained for 2.5 h at 80 °C. After cooling, the mixture was mixed with 5 mL 7% potassium carbonate solution and the supernatant was collected. The fatty acids of egg yolk were analyzed by a gas chromatograph (6890 series, Agilent Technologies, Wilmington, DE, USA) equipped with a capillary column (length 60 m, internal diameter 0.25 mm, film thickness 0.25 μm; DB-23, Agilent) and a flame ionization detector. Finally, fatty acids were expressed as the proportion of each individual fatty acid to the total amount of all fatty acids in the sample.

### Serum parameters

T-AOC (A015-3-1), CAT (A007-1-1), GSH-Px (A005-1), SOD (A001-3), glucose (GLU, A154-2-1), triglyceride (TG, A110-1-1), total cholesterol (TC, A111-1-1), total protein (TP, A045-4-2), albumin (ALB, A028-2-1), interleukin (IL)-1β (H002-1-2), IL-6 (H007-1-2), IL-10 (H009-1-2), and tumor necrosis factor-α (TNF-α, H052-1-2) were measured by colorimetric kits. All kits were purchased from Nanjing Jiancheng Bioengineering Institute (Jiangsu, China).

### Antioxidant status of the jejunal mucosa

The jejunal mucosa was homogenized in saline solution (1:9), centrifuged at 2,500 × *g* for 10 min, and the supernatant samples were collected for determining the antioxidant status of jejunal mucosa. T-AOC, CAT, GSH-Px, and SOD in the jejunal samples were measured by colorimetric kits (Nanjing Jiancheng Bioengineering Institute, China).

### Quantitative real-time PCR

Mucosal RNA was extracted from the jejunum using an EASYspin RNA Mini Kit (Aidlab Biotechnologies, Co., Ltd., Beijing, China). Reverse transcription was conducted using the HiScript III 1st Strand cDNA Synthesis Kit (Vazyme Biotech Co., Ltd., Jiangsu, China). Real-time PCR (RT-PCR) was carried out using the Mx3000P system (Agilent StrataGene). The expressions of targeted genes were obtained by the 2^−ΔΔCT^ method. Primers were shown in Table S2.

### Analysis of cecal short-chain fatty acids

The composition of short-chain fatty acids (SCFA) was detected based on the method of Zhang and Piao [16]. The content of SCFA in cecum was measured using a high-performance ion chromatograph (DIONEX ICS-3000, Thermo Fisher, Waltham, MA, USA). Cecal digesta (0.5 g) was mixed with 8 mL ultrapure water. After centrifugation, the supernatant was diluted (1:50) using ultrapure water and then filtered through 0.22 μm membrane (Jinlong, JY-B11090871) before injection into an AG11 guard column (250 mm × 4 mm) and an AG11 guard column using KOH for isocratic elution. The injection volume was 25 μL and the flow rate was 1.0 mL/min. The contents of SCFA were expressed as mg/g of the cecal digesta.

### Cecal microbial community

Bacterial DNA was extracted from cecal digesta using a Stool DNA Kit (Omega Bio-tek, Norcross, GA, USA). The DNA concentration was quantified by NanoDrop 2000 UV–Vis spectrophotometer (Thermo Scientific, Wilmington, USA), and the integrity of DNA was checked by 1% agarose gel. The V3–V4 regions of the microbial 16S rRNA gene were amplified using primers 338F (5'-ACTCCTRCGGGAGGCAGCAG-3') and 806R (5'-GGACTACCVGGGTATCTAAT-3'). Then the products were separated from 2% agarose gels and recovered using the AxyPrep DNA Gel Extraction Kit (Axygen Biosciences, Union City, CA, USA). The purified amplicons were pooled and paired-end sequenced on the Illumina MiSeq platform. The raw sequencing reads were demultiplexed, quality-filtered by fastp, and merged by FLASH to obtain high-quality effective tags with reference to the tags quality control process of QIIME (version 1.17). Then the rest high-quality sequences were clustered into operational taxonomic units (OTU) with a similarity of 97% using UPARSE software. The taxonomy of 16S rRNA gene sequences was determined by the RDP Classifier with a confidence greater than 70%. Principal coordinate analysis (PCoA) and non-metric multidimensional scaling (NMDS) based on the Bray–Curtis distance matrix algorithm were generated using the “ggplot2” packages of the R software (version 3.3.1). Linear discriminant analysis effect size (LEfSe) analysis combined with an all-against-all multi-group comparison strategy (LDA score > 2.0) on the basis of the nonparametric factorization Kruskal–Wallis sum test and Wilcoxon rank sum test was applied for estimating features with significant differences in abundance and identifying taxa with significant abundances. Metabolic functions of cecal microbiota were predicted by Phylogenetic Investigation of Communities by Reconstruction of Unobserved States (PICRUSt2).

### RNA-seq analysis and validation using RT-PCR

Total RNA of the oviductal magnum was isolated for sequencing using the Illumina NovaSeq 6000 platform (Illumina, San Diego, CA, USA). Sequence adapters and low-quality reads (read quality < 30) were removed using Trimmomatic [17]. Quality control checks on raw sequence data were performed with FastQC. Then, sequencing reads were mapped to the *Gallus_gallus* reference genome (GRCg6a) using HISAT2 [18]. Mapped reads were assembled by StringTie [19]. Gene expression levels were calculated as FPKM using RSEM software [20] and differentially expressed genes (DEGs) were determined using DESeq2 [21]. Gene expressions with a false discovery rate (FDR) adjusted *P*-value < 0.05 and |log_2_(fold change)|> 1 were considered significantly different Data processing were conducted using the Majorbio cloud platform (https://cloud.majorbio.com/). The expressions of selected DEGs were validated using RT-PCR. All primers for targeted genes are present in Table S2.

### Statistical analysis

The Shapiro–Wilk and Levene’s tests were used to verify the normal distribution and homogeneity of variances of the data. Data analysis was performed using one-way ANOVA via the GLM procedure of SAS 9.4 (SAS Inst. Inc., Cary, NC, USA), and statistical differences were compared using Tukey's tests. Each bird (pen) was considered as the experimental unit. Models included treatment as the fixed effect and replicate as the random effect. Differences in the intestinal microbiota were analyzed by Kruskal–Wallis rank sum test with Tukey–Kramer post hoc test and Benjamini–Hochberg false discovery rate. The correlations among cecal microbiota, SCFA, egg quality, and serum parameters were analyzed by Spearman correlation analysis. The correlation coefficient ranges from −1 to 1, and the greater the absolute value, the stronger the correlation. Differences in the predictive metabolic functions were analyzed by STAMP using Welch’s *t*-test. *P*-value < 0.05 was considered a significant difference, and 0.05 ≤ *P* < 0.10 reflected a tendency.

## Results

### Performance and egg quality

Table 1 showed no significant differences for performance. On d 56, birds in the RE200 group had higher (*P* < 0.05) Haugh unit than the CON group (Table 2). However, no difference was observed for Haugh unit between CTC and RE200 groups.

**Table 1:** Effects of rosemary extract on laying performance of laying hens^1^

Item	CON	CTC	RE50	RE100	RE200	SEM	*P*-value
Before the formal experiment							
Egg production, %	79.17	79.37	78.57	79.37	78.57	2.29	0.99
1–4 weeks							
Egg production, %	74.06	80.78	79.51	82.74	81.47	4.22	0.63
Egg weight, g	57.39	58.08	57.62	56.99	58.72	0.65	0.41
Egg mass, g/d per bird	44.56	46.98	45.99	47.13	45.95	2.60	0.96
Average daily feed intake, g/d per bird	107.86	107.91	103.75	108.07	105.88	2.40	0.66
Feed conversion ratio, g/g	2.51	2.31	2.37	2.30	2.28	0.19	0.89
5–8 weeks							
Egg production, %	75.82	80.88	78.83	82.02	78.61	2.87	0.59
Egg weight, g	58.33	57.87	58.31	58.06	60.00	0.65	0.18
Egg mass, g/d per bird	45.22	46.97	46.23	47.88	48.14	2.11	0.86
Average daily feed intake, g/d per bird	108.13	105.46	108.94	106.13	103.96	1.78	0.32
Feed conversion ratio, g/g	2.45	2.25	2.38	2.22	2.19	0.11	0.47
1–8 weeks							
Egg production, %	74.94	80.83	79.17	82.38	80.04	3.29	0.57
Egg weight, g	57.86	57.98	57.96	57.52	59.36	0.63	0.31
Egg mass, g/d per bird	44.89	46.97	46.11	47.51	47.14	2.23	0.92
Average daily feed intake, g/d per bird	107.99	106.68	106.34	107.10	104.50	1.88	0.78
Feed conversion ratio, g/g	2.47	2.28	2.35	2.26	2.23	0.13	0.70

*CON* Control, *CTC* 50 mg/kg chlortetracycline, *RE50* 50 mg/kg rosemary extract, *RE100* 100 mg/kg rosemary extract, *RE200* 200 mg/kg rosemary extract

^1^Values are shown as mean ± SEM, *n* = 6

**Table 2:** Effects of rosemary extract on egg quality of laying hens^1^

Item	CON	CTC	RE50	RE100	RE200	SEM	*P*-value
Yolk color	7.56	7.61	7.11	6.94	8.28	0.38	0.15
Albumen height, mm	4.37	4.49	4.21	4.51	4.75	0.22	0.53
Haugh unit	58.51^b^	63.07^ab^	61.87^ab^	64.10^ab^	66.32^a^	1.69	0.04
Eggshell strength, kgf	3.84	3.66	3.75	3.88	3.84	0.15	0.84
Eggshell thickness, mm	0.31	0.33	0.32	0.31	0.32	0.01	0.32

*CON* Control, *CTC* 50 mg/kg chlortetracycline, *RE50* 50 mg/kg rosemary extract, *RE100* 100 mg/kg rosemary extract, *RE200* 200 mg/kg rosemary extract

^1^Values are shown as mean ± SEM, *n* = 6

^a,b^Results without a common superscript means significant difference at *P* < 0.05

### Fatty acid profile of egg yolk

As shown in Table 3, birds in the RE100 and RE200 groups had significantly higher contents of C18:0 and C18:1 n-9c than the CON group. In comparison with the CON group, n-6/n-3 significantly reduced and n-6 of egg yolk tended to increase in the RE200 group. However, no significant differences were detected for C15:0, C18:0, C18:1 n-9c, and n-6/n-3 between the CTC and RE200 groups.

**Table 3:** Effects of rosemary extract on fatty acid profile (g/100 g) in egg yolk of laying hens^1^

Item	CON	CTC	RE50	RE100	RE200	SEM	*P*-value
C12:0	0.003	0.003	0.003	0.003	0.003	0.000	0.47
C14:0	0.19	0.17	0.18	0.17	0.19	0.001	0.53
C14:1	0.05	0.04	0.04	0.04	0.04	0.004	0.22
C15:0	0.020^a^	0.017^ab^	0.012^c^	0.013^bc^	0.014^bc^	0.001	< 0.01
C16:0	13.26	12.78	13.05	12.88	13.29	0.26	0.56
C16:1	1.79	1.64	1.64	1.65	1.56	0.12	0.72
C18:0	4.18^b^	4.37^ab^	4.50^ab^	4.94^a^	5.03^a^	0.16	< 0.01
C18:1 n-9c	10.55^c^	10.60^bc^	11.10^abc^	11.36^a^	11.31^ab^	0.21	0.02
C18:2 n-6c	7.96	6.99	6.97	7.00	7.71	0.32	0.09
C18:3 n-3	0.28	0.25	0.24	0.26	0.29	0.02	0.20
C20:2	0.12	0.10	0.11	0.12	0.12	0.005	0.07
C21:0	0.03	0.03	0.04	0.04	0.04	0.003	0.06
C20:3 n-6	0.10	0.08	0.10	0.09	0.09	0.007	0.53
C20:4 n-6	1.09	1.13	1.11	1.19	1.21	0.04	0.23
C22:6 n-3	0.55	0.62	0.62	0.64	0.70	0.04	0.11
SFA	17.68	17.37	17.77	18.04	18.56	0.37	0.24
MUFA	12.39	12.29	12.78	13.05	12.92	0.23	0.10
PUFA	10.10	9.17	9.16	9.30	10.12	0.36	0.14
n-6	9.16	8.20	8.18	8.29	9.00	0.34	0.13
n-3	0.83	0.87	0.86	0.90	1.00	0.04	0.09
n-6/n-3	11.14^a^	9.46^ab^	9.61^ab^	9.37^ab^	9.06^b^	0.49	0.04
UFA/SFA	1.27	1.24	1.24	1.24	1.25	0.03	0.91

*CON* Control, *CTC* 50 mg/kg chlortetracycline, *RE50* 50 mg/kg rosemary extract, *RE100* 100 mg/kg rosemary extract, *RE200* 200 mg/kg rosemary extract

^1^Values are shown as mean ± SEM, *n* = 6

^a–c^Results without a common superscript means significant difference at *P* < 0.05

### Serum parameters

**Fig. 1:**
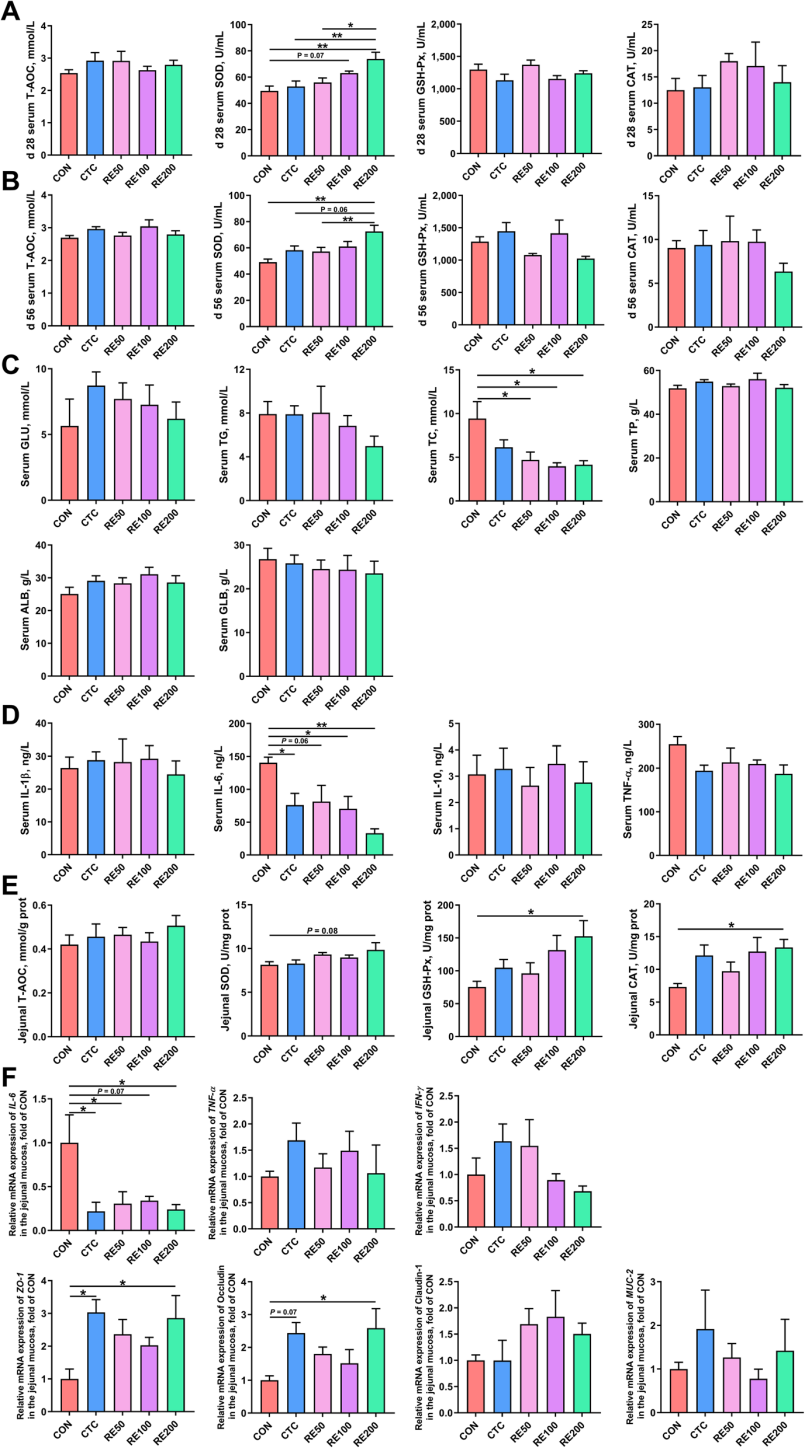
Effects of rosemary extract on serum parameters and intestinal function of laying hens. Serum antioxidant status at d 28 (**A**) and d 56 (**B**). **C** Serum inflammatory cytokines. **D** Serum biochemical parameters. **E** Antioxidant status in the jejunal mucosa. **F** The relative mRNA expression of genes related to intestinal immunity and barrier function in the jejunal mucosa. CON, control; CTC, 50 mg/kg chlortetracycline; RE50, 50 mg/kg rosemary extract; RE100, 100 mg/kg rosemary extract; RE200, 200 mg/kg rosemary extract. Values are shown as mean ± SEM, *n* = 6. ^*^P < 0.05; ^**^P < 0.01

On d 28, birds in the RE200 group had significantly higher serum SOD activity than those in the CON, CTC and RE50 groups (Fig. 1A). RE100 tended to increase (*P* = 0.07) serum SOD activity than that in the CON group. On d 56, RE200 significantly enhanced serum SOD activity compared to the CON and RE50 groups, and tended to improve (*P* = 0.06) serum SOD compared to the CTC group (Fig. 1B). As shown in Fig. 1C, birds in the CTC, RE100 and RE200 groups had significantly lower serum IL-6 level than the CON group. In addition, RE treatment significantly decreased serum TC content than that in the CON group (Fig. 1D).

### Intestinal antioxidant capacity and barrier function

In comparison with the CON group, jejunal SOD activity tended to enhance (*P* = 0.08) and jejunal activities of GSH-Px and CAT markedly enhanced in the RE200 group (Fig. 1E). As shown in Fig. 1F, birds in the RE200 and CTC groups had lower expression of jejunal *IL-6* and higher abundance of jejunal *ZO-1* than the CON (*P* < 0.05). In comparison with the CON, the abundance of jejunal *Occludin* markedly enhanced in the RE200 group and the expression of jejunal *Occludin* tended to enhance (*P* = 0.07) in the CTC group.

### SCFA profile of cecal digesta

**Fig. 2:**
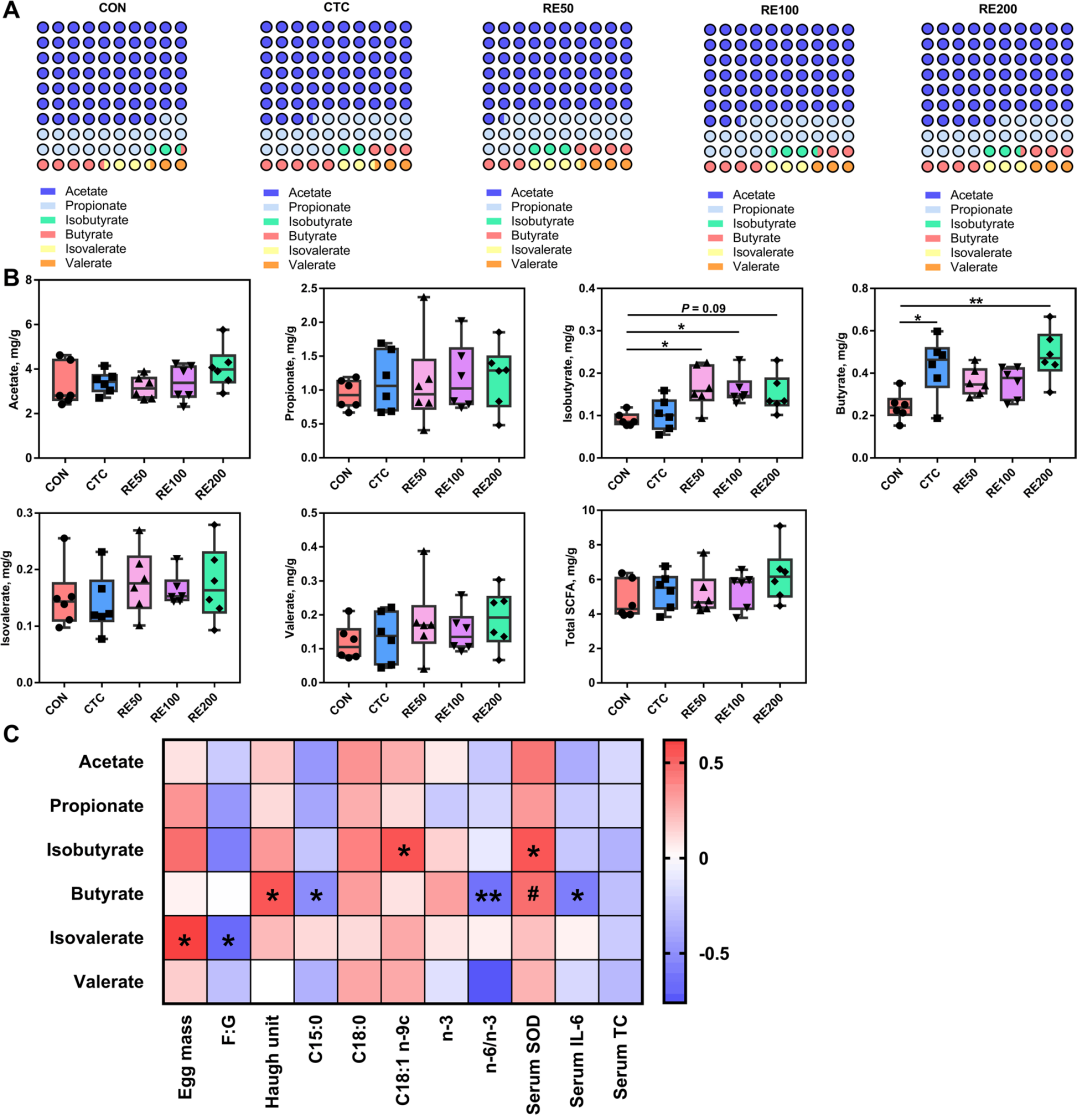
Effects of rosemary extract on compositional proportion and contents of cecal SCFA in laying hens. **A** SCFA compositional proportion. **B** SCFA contents. **C** Spearman correlation heatmap between cecal SCFA, egg quality, and serum parameters. CON, control; CTC, 50 mg/kg chlortetracycline; RE50, 50 mg/kg rosemary extract; RE100, 100 mg/kg rosemary extract; RE200, 200 mg/kg rosemary extract. Values are shown as mean ± SEM, *n* = 6. ^#^0.05 ≤ *P* < 0.1; ^*^P < 0.05; ^**^P < 0.01

The compositional proportion and concentrations of SCFA in the cecal digesta are shown in Fig. 2. Among all treatments, the composition of SCFA in the cecal digesta varied greatly (Fig. 2A). Birds in the RE50 and RE100 groups had markedly higher cecal isobutyrate than the CON, and RE200 tended to increase (*P* < 0.05) cecal isobutyrate content compared to the CON (Fig. 2B). After supplemented with CTC and RE200, cecal butyrate content markedly enhanced when compared with the CON group. As shown in Fig. 2C, butyrate content was positively associated with Haugh unit (*P* < 0.05; *r* = 0.547) and serum SOD activity (*P* = 0.06; *r* = 0.470), and negatively (*P* < 0.05) associated with n-6/n-3 (*r* =  −0.645) and serum IL-6 (*r* =  −0.584) (Fig. 2C). In addition, cecal isobutyrate content was positively (*P* < 0.05) associated with C18:1 n-9c (*r* = 0.559) and serum SOD activity (*r* = 0.550).

### Cecal microbial structure and community

**Fig. 3:**
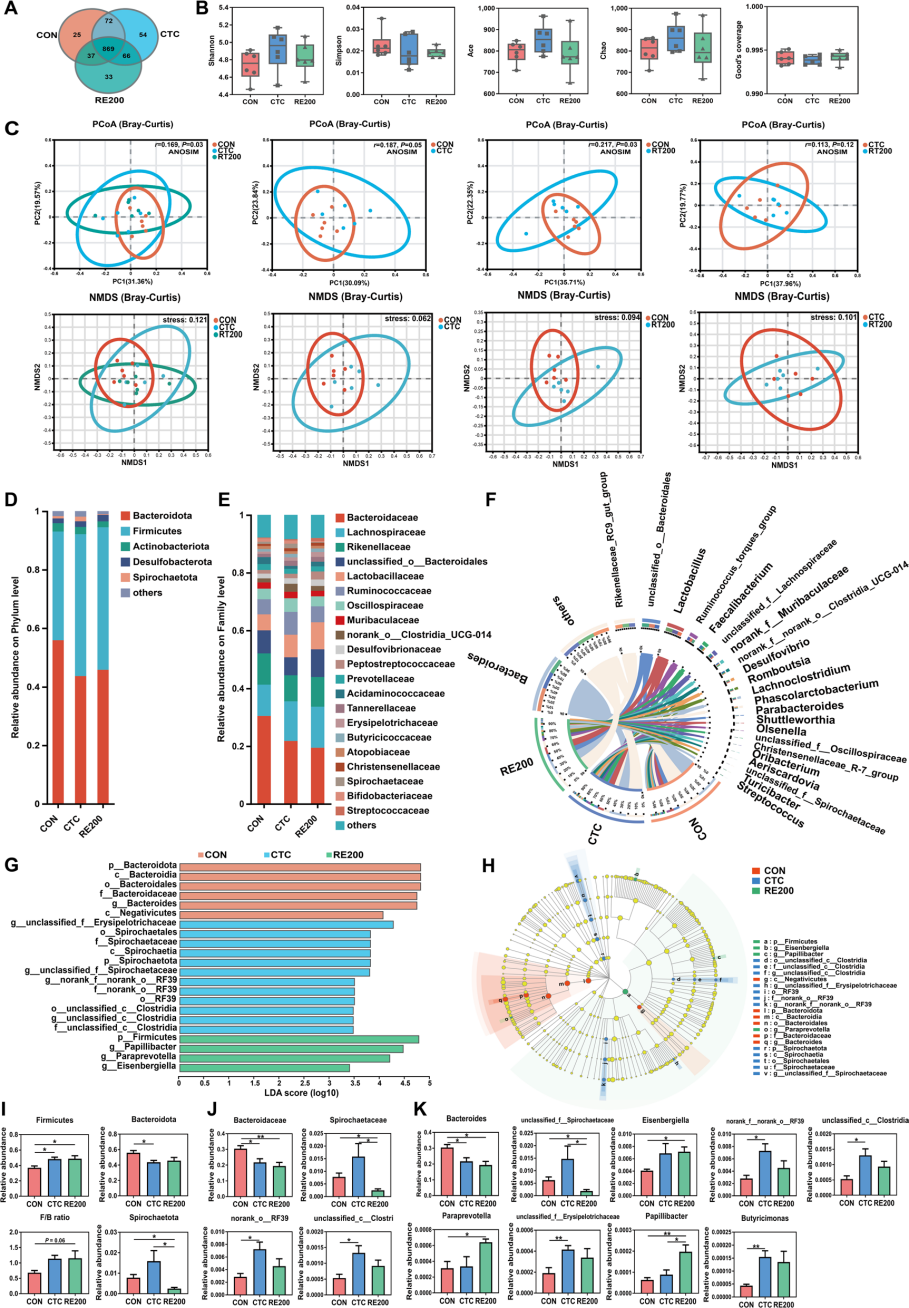
Effects of rosemary extract on cecal microbiota structure and composition of laying hens. **A** OUT Venn. **B** Comparison of α-diversity indices. **C** Comparison of β-diversity indices. **D** Microbiota composition at the phylum level. **E** Microbiota composition at the family level. **F** Microbiota composition at the genus level. **G** Linear discriminant analysis (LDA) distribution, and the score > 2 means significant. **H** Cladogram of LEfSe shows taxonomic profiling from the phylum to genus level, the yellow node represents no difference, but other color nodes represent significant difference. **I** Differences in the cecal microbiota composition at the phylum level. **J** Differences in the cecal microbiota composition at the family level. **K** Differences in the cecal microbiota composition at the genus level. CON, control; CTC, 50 mg/kg chlortetracycline; RE200, 200 mg/kg rosemary extract. Values are shown as mean ± SEM, *n* = 6. ^*^P < 0.05; ^**^P < 0.01

The cecal contents collected from the CON, CTC and RE200 groups were used to determine microbial structure and community. Figure 3A showed that 47, 34 and 63 unique OTU were identified in the CON, CTC and RE200 groups, respectively. The α-diversity analysis showed no differences among treatments (Fig. 3B). PCoA analysis showed that cecal microbial community structure changed with treatments (Fig. 3C). The results revealed that CTC and RE200 significantly altered β-diversity index of cecal microbial community compared with the CON group. However, the β-diversity analysis showed no significant difference between the CTC and RE200 groups.

The dominant phyla were Bacteroidota and Firmicutes, accounting for 90% (Fig. 3D). Down to the family level, the predominant bacteria were Bacteroidaceae, Lachnospiraceae, Rikenellaceae, unclassified_o__Bacteroidales, Lactobacillaceae, Ruminococcaceae, Oscillospiraceae, Muribaculaceae, norank_o__Clostridia_UCG-014, Desulfovibrionaceae and so on (Fig. 3E). At the genus level, a circus diagram (Fig. 3F) showed the composition of cecal microbiota. The predominant genera were *Bacteroides*, *unclassified_o__Bacteroidales*, *Lactobacillus*, *Ruminococcus_torques_group*, *Rikenellaceae_RC9_gut_group*, *Faecalibacterium*, *unclassified_f__Lachnospiraceae*, *norank_f__Muribaculaceae*, *Romboutsia*, *Desulfovibrio*, and so on. The specific bacterial taxa associated with treatments was identified by LEfSe (LDA score > 2) analysis. Our results revealed 22 different bacterial taxa among three treatments (Fig. 3G). Among these bacterial taxa, 6 of bacterial taxa were characteristic for the CON group, 12 of bacterial taxa were characteristic for the CTC group, and 4 of bacterial taxa were characteristic for the RE200 group. A large abundance of Bacteroidota, Bacteroidia, Bacteroidales, Bacteroidaceae, *Bacteroides*, Negativicutes in the CON group, *unclassified_f__Erysipelotrichaceae*, Spirochaetales, Spirochaetaceae, Spirochaetia, Spirochaetota, *norank_f__norank_o__RF39*, *unclassified_f__Spirochaetaceae*, norank_o__RF39, RF39, *unclassified_c__Clostridia*, in the CTC group, Firmicutes, *Papillibacter*, *Paraprevotella*, *Eisenbergiella* in the RE200 group were detected (Fig. 3H). The different bacteria were further determined by the Kruskal–Wallis rank sum test (Fig. 3I–K). The Firmicutes abundance was higher (*P* < 0.05) and Bacteroidaceae and *Bacteroides* in the CTC and RE200 were lower (*P* < 0.05) compared to the CON. Spirochaetota, Spirochaetaceae and *unclassified_f__Spirochaetaceae* in the cecal digesta of the RE200 was reduced (*P* < 0.05) compared to the CON and CTC. RE200 enhance (*P* = 0.06) the ratio of Firmicutes to Bacteroidota and markedly enhanced the abundance of *Eisenbergiella* and *Paraprevotella* compared to the CON. Compared to the CON and CTC groups, the abundance of *Papillibacter* in the RE200 was significantly increased. The abundance of norank_o__RF39, *unclassified_c__Clostridia*, *norank_f__norank_o__RF39*, *Butyricimonas*, and *unclassified_f__Erysipelotrichaceae* in the CTC was markedly enhanced (*P* < 0.05) compared to the CON.

### The correlations among differential cecal microbiota, SCFA, egg quality and serum parameters

**Fig. 4:**
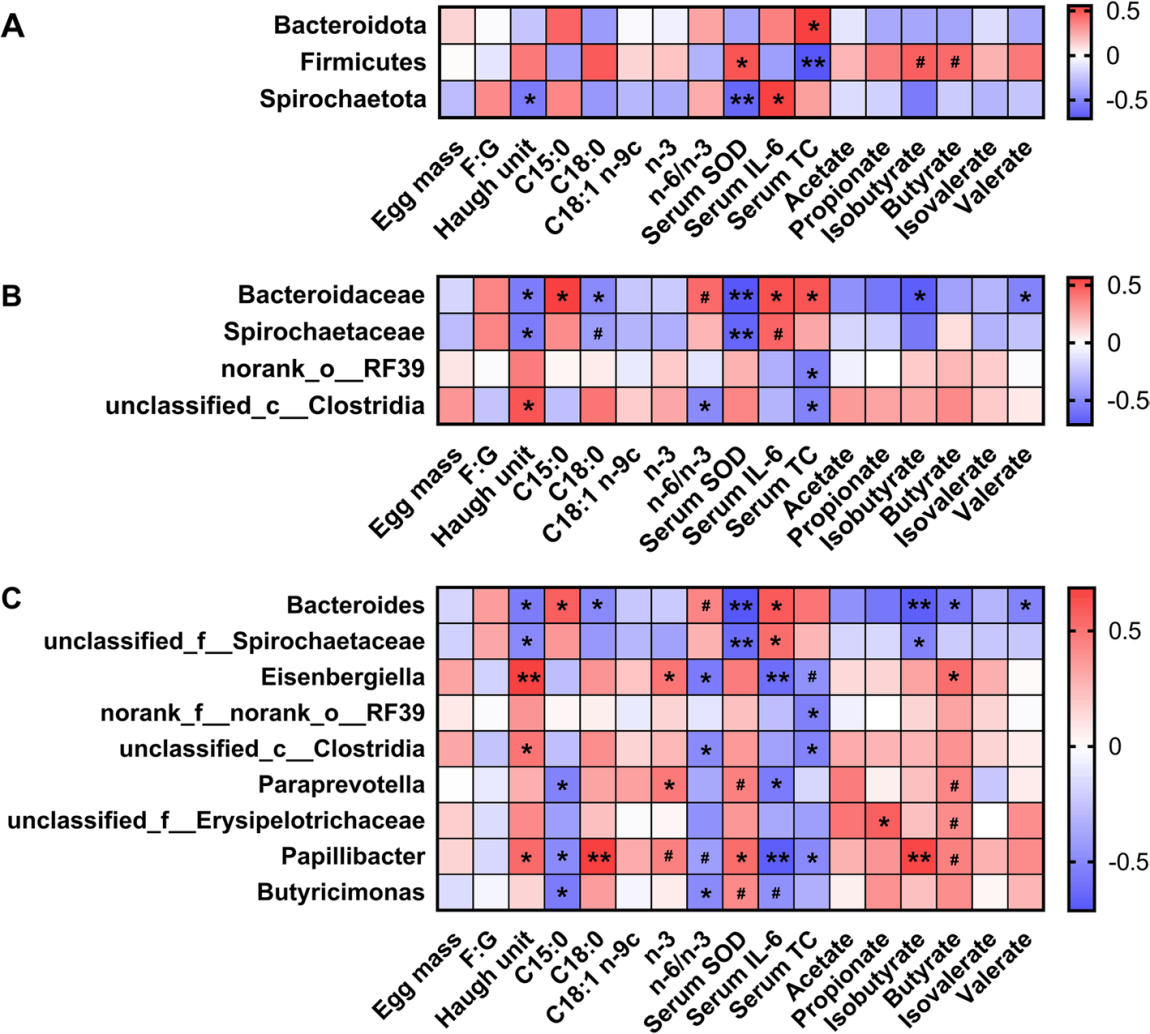
Heatmap of Spearman’s correlations among SCFA, egg quality, serum parameters, and differential cecal microbiota at the phylum (**A**), family (**B**), and genus (**C**) levels. ^#^0.05 ≤ *P* < 0.1; ^*^P < 0.05; ^**^P < 0.01

For differential phylum (Fig. 4A), Firmicutes in the cecal digesta was positively (*P* < 0.05) associated with serum SOD and tended to be positively associated with cecal contents of isobutyrate and butyrate. Serum TC level was negatively associated with Firmicutes and positively associated with Bacteroidota (*P* < 0.05). Spirochaetota in the cecal digesta was positively associated with serum IL-6 level and negatively related to Haugh unit and serum SOD (*P* < 0.05). For differential bacteria at the family level (Fig. 4B), the Bacteroidaceae abundance was positively associated with serum IL-6 and TC and negatively related to Haugh unit, C18:0, serum SOD and isobutyrate (*P* < 0.05). Spirochaetaceae was negatively (*P* < 0.05) associated with Haugh unit and serum SOD. For differential bacteria at the genus level (Fig. 4C), the *Bacteroides* abundance was positively associated with serum IL-6 level and negatively associated with Haugh unit, C18:0, serum SOD, isobutyrate, and butyrate (*P* < 0.05). *Eisenbergiella* was positively associated with Haugh unit, n-3, and butyrate and negatively associated with n-6/n-3 and serum TC level (*P* < 0.05). *Paraprevotella* was positively associated with n-3, serum SOD, and butyrate and negatively (*P* < 0.05) related to serum IL-6 level. The *Papillibacter* abundance was positively associated with Haugh unit, C18:0, n-3, serum SOD, isobutyrate and butyrate and negatively associated with n-6/n-3, and serum levels of IL-6 and TC. The *Butyricimonas* abundance was positively related to serum SOD and negatively related to n-6/n-3, and serum IL-6 level.

### Functional prediction on carbohydrate and amino acid metabolism of cecal microbiota

**Fig. 5:**
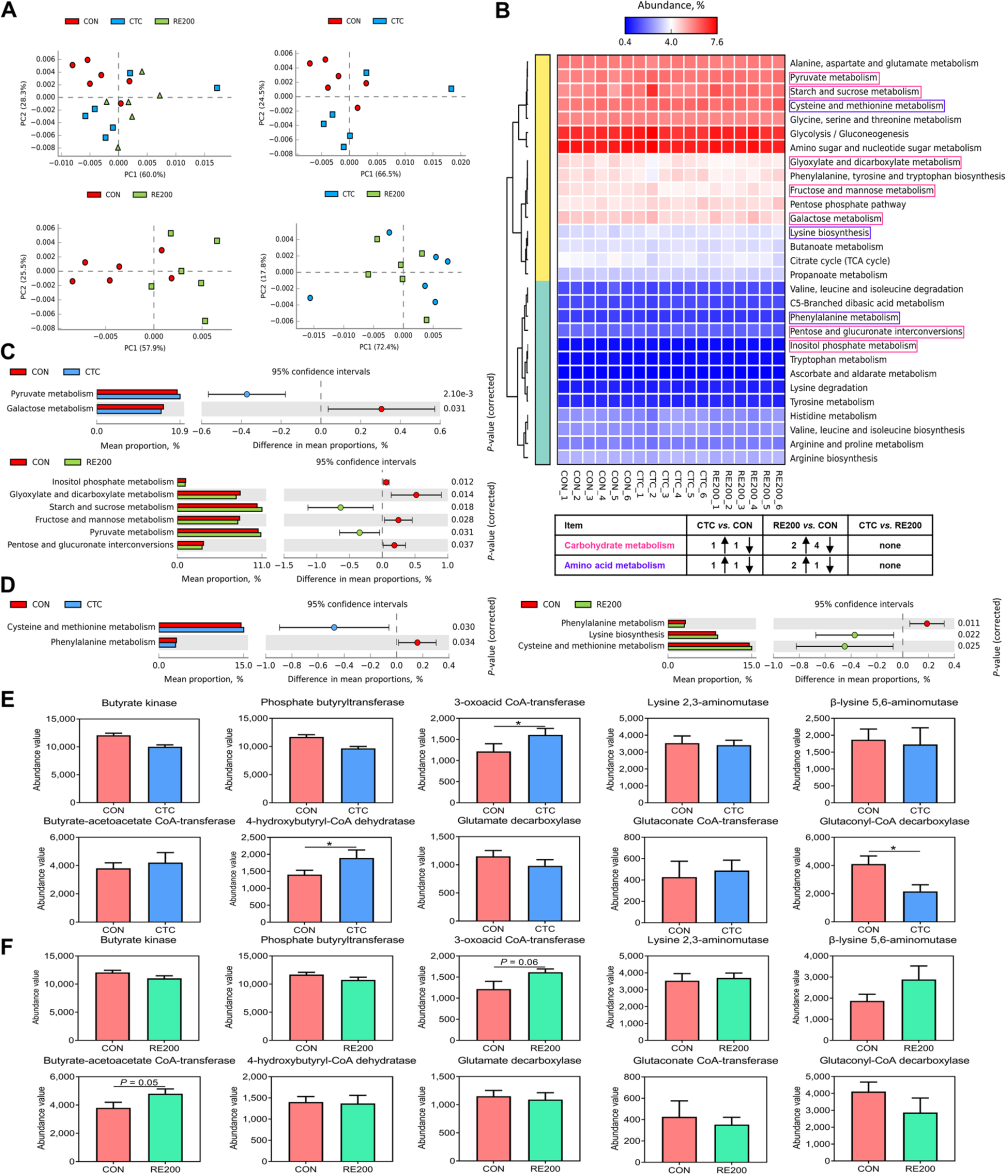
Functional prediction on carbohydrate and amino acid metabolism of cecal microbiota using PICRUSt2. **A** PCA analysis of KEGG function. **B** Relative abundance of functional prediction on carbohydrate and amino acid metabolism. **C** Differences in the functional prediction on carbohydrate metabolism. **D** Differences in the functional prediction on amino acid metabolism. **E** Abundance values of butyrate synthesizing enzymes in the cecal microbiota between CON and CTC groups. **F** Abundance values of butyrate synthesizing enzymes in the cecal microbiota between CON and RE200 groups. CON, control; CTC, 50 mg/kg chlortetracycline; RE200, 200 mg/kg rosemary extract. Values are shown as mean ± SEM, *n* = 6. ^*^P < 0.05

To predict metabolic functions, PICRUSt2 predictions of function were obtained based on the KEGG database. PCA analysis revealed that greater variations were observed for metabolic functions of cecal microbiota in the CTC and RE200 compared to the CON, and the CTC and RE200 had similar metabolic functions of cecal microbiota (Fig. 5A). A shown in Fig. 5B, four different functional genes related to carbohydrate and amino acid metabolism between CON and CTC groups were observed, and nine different functional genes related to carbohydrate and amino acid metabolism between CON and RE200 groups were observed. However, no significant differences were detected for functional genes related to carbohydrate and amino acid metabolism between CTC and RE200 groups. As shown in Fig. 5C and D, compared to the CON, the expression of function genes related to pyruvate metabolism and cysteine and methionine metabolism significantly increased in the CTC group, whereas the expression of function genes related to galactose metabolism and phenylalanine metabolism markedly reduced in the CTC. In comparison with the CON group, the expression of function genes related to starch and sucrose metabolism, pyruvate metabolism, cysteine and methionine metabolism, and lysine biosynthesis markedly enhanced in the RE200 group, whereas the expression of function genes related to fructose and mannose metabolism, inositol phosphate metabolism, glyoxylate and dicarboxylate metabolism, phenylalanine metabolism, and pentose and glucuronate interconversions significantly decreased in the RE200 group. Figure 5E and F showed the abundance values of function genes related to butyrate synthesizing enzymes. Compared to the CON, the abundance values of 3-oxoacid CoA-transferase and 4-hydroxybutyryl-CoA dehydratase markedly enhanced in the CTC group, and the abundance values of 3-oxoacid CoA-transferase (*P* = 0.06) and butyrate-acetoacetate CoA-transferase (*P* = 0.05) tend to be higher in the RE200 group.

### Functional metabolism profiling of oviductal magnum

**Fig. 6:**
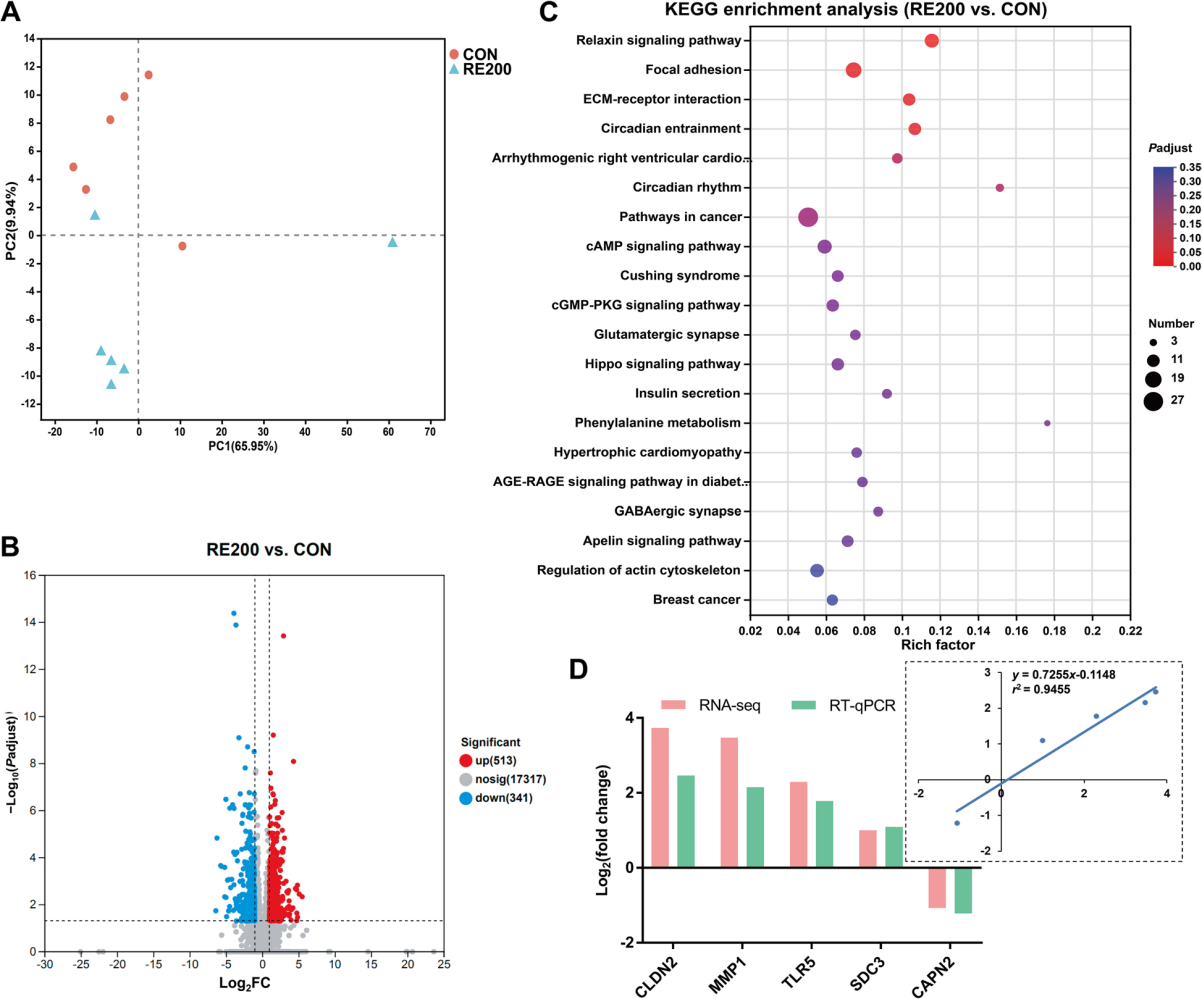
RNA-seq analysis in the oviductal magnum and RT-PCR validation of gene expression. **A** PCA analysis. **B** A Volcano plot of differentially expressed genes. **C** Dot plot of KEGG enrichment analysis of differentially expressed genes. **D** Fold change of selected genes by RNA-seq and the correlation analysis of selected genes between the RNA-seq and RT-PCR results. CON, control; RE200, 200 mg/kg rosemary extract, *CLDN2*, claudin-2; *MMP1*, matrix metallopeptidase 1; *TLR5*, toll like receptor 5; *SDC3*, syndecan 3; *CAPN2*, calpain 2. *n* = 6

The yield, purity, and RIN value of RNA extracted from oviductal magnum are shown in Tables S3. Table S4 presents number of reads derived from the RNA-seq analysis, which were mapped successfully to the *Gallus_gallus* reference genome (GRCg6a). PCA analysis showed the transcriptomic profile in the oviductal magnum with a clear separation of samples from the CON and RE200 groups (Fig. 6A). 854 DEGs were observed between the CON and RE200 groups, with 513 were markedly enhanced (*P* < 0.05) and 341 were markedly reduced (*P* < 0.05) in the RE200 group (Fig. 6B). KEGG analysis demonstrated four pathways that were markedly enriched with DEGs, such as relaxin signaling pathway, focal adhesion, ECM-receptor interaction, and circadian entrainment (Fig. 6C). The top 75 up- and down-regulated DEGs are shown in Tables S5 and S6. We selected genes involved in tight junction proteins (*CLDN2*), immune function (*TLR5*), and albumen formation (*MMP1*, *SDC5*, *CAPN2*) validate using RT-qPCR analysis (Fig. 6D). Validation of selected genes using RT-PCR revealed that the gene expressions of *CLDN2*, *TLR5*, *MMP1*, and *SDC5* were enhanced and the gene expression of *CAPN2* was reduced in the RE200 group compared to the CON. The results showed a good correlation (*R*^2^ = 0.9455) between RT-PCR and the RNA-seq data.

## Discussion

Egg internal quality is important to poultry production and human health. The egg-white quality and fatty acid profile of yolk are of great importance for customer preference. The Haugh unit was obtained based on the weight of egg and the thickness of egg-white [1], which is considered as a key parameter for evaluating albumen quality and associated with shelf life. RE at 200 mg/kg enhanced Haugh unit compared to the CON, but no difference was detected for Haugh unit between CTC and RE200 groups. Similar findings were reported [1], which suggested that the addition of antioxidant (tea polyphenol) to diets enhanced Haugh unit of late-phase laying hens. Importantly, optimal n-6/n-3 in food was beneficial to human health.

As the most affordable protein source, there is increasing preference among individuals to consume eggs with higher content of n-3 PUFA, especially C22:6 n-3 and C20:5 n-3. These essential fatty acids could lower triglycerides concentration, stabilize membrane structure, and they are believed to have antithrombotic, anti-inflammatory, and antiarrhythmic properties [22]. High n-6/n-3 pushes the pathogenesis of several diseases, such as autoimmune disease, osteoporosis, and cardiovascular disease, but lower n-6/n-3 (or higher n-3 PUFA) exerts suppressive effects on the above diseases [23]. Several fatty acids, including C22:6 n-3, C18:1 n-9c, and C18:3 n-3, are considered flavor precursors, and their contents can be responsible for the rich aroma of animal-derived foods [24]. Our research demonstrated that 200 mg/kg RE improved n-6/n-3 of yolk in late-phase laying hens compared to the CON, but no difference was detected for n-6/n-3 of yolk between the CTC and RE200 groups. In addition, compared to the CON, RE at 100 and 200 mg/kg enhanced C18:1 n-9c content of egg yolk. Therefore, the present study suggested that RE could be considered a promising feed additive for improving specific egg quality characteristics associated with consumer acceptability. We speculated that these beneficial functions may be due to the active ingredients of RE used in this study, which have been reported to play important roles in antioxidant [3] and anti-inflammatory [4], as well as regulating intestinal barrier and microbiota and [11, 12].

Serum biochemical parameters are indicators of the internal status of laying hens. Serum TC was associated with lipid metabolism in the liver, which was markedly increased in fatty liver-laying hens [25]. Dietary RE supplementation significantly decreased serum TC level compared with the CON group, suggesting that RE could improve lipid metabolism and alleviate fatty liver disease of laying hens. The antioxidant status in serum can reflect the resistance to oxidative damage, and higher level of antioxidant ability can efficiently relieve oxidative damage [26]. Moreover, oxidative damage has negative effects on mammalian tissue, especially the intestines [27]. In this study, RE200 increased serum SOD activity than those fed with the CON and CTC diets. Moreover, RE200 tended to increase jejunal SOD activity and markedly enhanced jejunal activities of CAT and GSH-Px compared to the CON. SOD is the first line of defense against excessive oxidative radicals and can catalyze the conversion of superoxide radicals to H_2_O_2_, which is broken down by GSH-Px and CAT into H_2_O and O_2_. The results suggested the potential of RE supplementation for scavenging excessive ROS production and alleviating mucosal oxidative injury in laying hens. The inflammatory cytokines (IL-1β, TNF-α, and IL-6, etc.) can modulate inflammatory response and impair tight junction of the intestine [28]. Our results showed that RE200 and CTC groups decreased serum IL-6 level and enhanced the abundances of jejunal *ZO-1* and *Occludin* compared to the CON, indicating that lower serum pro-inflammatory cytokines in the RE200 and CTC groups may be part of the reason for the improvement in gut tight junction of laying hens. Collectively, RE at 200 mg/kg increased the performance and egg quality of laying hens partially by alleviating intestinal oxidative injury and enhancing gut barrier function.

The fermentation of carbohydrates and protein in diets produces SCFA, which can alleviate inflammatory response and prevent the imbalance of gut microbiota [29]. SCFA, especially butyrate, promotes the proliferation of *Lactobacillus* in poultry, which can further improve SCFA production, alleviate oxidative damage and inflammation in the gut, and suppress the growth of pathogenic bacteria such as *E. coli* and *Salmonella* [30]. Our study suggested that RE200 and CTC significantly increase cecal butyrate level, which may be partly contributed to the improvements in immunity and barrier function in laying hens. Spearman correlation also revealed that cecal butyrate level was positively associated with serum SOD and negative associated with serum IL-6 level, highlighting the beneficial effects of higher butyrate content induced by RE200 supplementation on intestinal barrier function. Based on the egg quality, serum parameters and cecal butyrate content, we focused on microbial community among the CON, CTC, and RE200 groups. Both diets and additives can regulate the composition of gut microbiota, while the alternation of the microbiota composition also influences the host’s digestion and utilization of the diets [31]. The present study revealed that the β-diversity index in the RE200 and CTC groups was markedly different from the CON group, indicating that dietary supplementation with RE200 and CTC markedly changed cecal microbial community structure. The most dominant bacteria at the phylum level were Bacteroidota and Firmicutes, which was consistent with the results reported by Lucke et al. [32]. Firmicutes and higher F/B ratio in the cecal bacteria can be beneficial for energy utilization and growth performance [33], and higher abundance of Firmicutes, belonging to butyrate-producing bacteria, is closely related to anti-inflammatory response. This study indicated that RE200 and CTC increased cecal Firmicutes abundance and RE200 tended to increase F/B ratio compared with the CON group, indicating that RE could improve intestinal microbial composition and maintain gut homeostasis of laying hens. Moreover, Spearman correlation also revealed that cecal Firmicutes abundance was positively related to serum SOD activity and negatively related to serum TC level. Down to the genus level, RE200 reduced the abundance of *Bacteroides* and *unclassified_f__Spirochaetaceae* and enhanced the relative abundance of *Eisenbergiella*, *Paraprevotella*, and *Papillibacter*, as well as CTC decreased the abundance of *Bacteroides* and enhanced the abundance of *unclassified_c__Clostridia*, *unclassified_f__Erysipelotrichaceae*, *norank_f__norank_o__RF39*, and *Butyricimonas* compared to the CON. *Bacteroides* has been reported to be positively associated with the gene expression of intestinal pro-inflammatory cytokines in laying hens, which could impair gut barrier function [34]. *Eisenbergiella* can play an important role in the synthesis of butyric acid, which is beneficial to the growth of intestinal epithelial cells [35]. *Paraprevotella*, *Papillibacter* and *Butyricimonas* were associated with butyrate generation in the intestine, which was favorable to performance of chickens [36–38]. Moreover, *unclassified_c__Clostridia* has been implicated in the biosynthesis of SCFA [39]. Our study further demonstrated that Firmicutes, *Eisenbergiella*, *Paraprevotella*, u*nclassified_f__Erysipelotrichaceae*, and *Papillibacter* were positively related to butyrate and Bacteroides was negatively related to butyrate. The results also explained why cecal butyrate content in the CTC and RE200 was higher than the CON group. And the correlation analysis further demonstrated that Firmicutes, Bacteroides, *Eisenbergiella*, *Paraprevotella*, *Papillibacter* and *Butyricimonas* were closely associated with serum contents of SOD, IL-6, and TC, indicating that RE and CTC significantly regulated the structure of cecal microbiota to more efficiently enhance the antioxidant ability and increase immune response of laying hens. Predictive metabolic functions of cecal microbiota by PICRUSt2 were analyzed by STAMP software. Our study showed that dietary RE and CTC supplementation significantly altered carbohydrate metabolism and amino acid metabolism, which may contribute to explaining changes in cecal SCFA content, especially butyrate, of laying hens. In order to prove this point, the abundances of butyrate synthesizing enzymes were further analyzed. Interestingly, the abundances of 3-oxoacid CoA-transferase and 4-hydroxybutyryl-CoA dehydratase markedly enhanced in the CTC group and the abundances of 3-oxoacid CoA-transferase and butyrate-acetoacetate CoA-transferase enhanced in the RE200 group compared to the CON, which directly led to the change in cecal butyrate concentration of laying hens.

Accumulating evidence demonstrated that gut microbiota can play a potential regulatory role on alleviating egg quality reduction in late-phase laying hens [40–42]. In addition to directly influencing egg quality by the vertical transmission route of gut-oviduct-egg, gut bacteria and metabolites, including SCFA, are indirectly participated in modulating egg quality via the microbiota-intestine-liver/brain-reproductive tract axis [43]. SCFAs can interact with intrinsic enteric neurons and intestine-innervating vagal and spinal afferents to influence the secretion of estradiol, which further modulates the formation of albumen in the oviducal magnum and finally achieves the improvements in egg-white quality. Moreover, intestinal microbiota can regulate fatty acid composition of yolk in Japanese quail, including increasing C18:0 and decreasing C14:1 and C16:1 [44]. It is worth noting that the addition of sodium butyrate to diets enhanced production performance and egg quality of laying hens such as yolk color and egg shell strength [45, 46]. In this study, spearman correlation between SCFA and egg quality indicated that butyrate significantly increased Haugh unit and decreased n-6/n-3 in egg yolk. It can also be seen that Firmicutes, *Eisenbergiella*, *Paraprevotella*, and *Papillibacter*, acting as butyrate-producing bacteria were also closely related to Haugh unit, n-3 and n-6/n-3 of yolk. Overall, the resulting changes in cecal microbiota induced by RE altered the content of intestinal SCFA, especially butyrate, which potentially influenced albumen quality and fatty acid deposition in egg yolk.

Egg albumen is synthesized in the oviductal magnum, which is a major factor related to interior egg quality. Given the increased Haugh unit induced by RE in this study, we further detected the transcriptomic profiling of oviductal magnum between the CON and RE200 groups. RNA-seq analysis demonstrated that 513 DEGs were significantly enhanced and 341 DEGs were markedly lower in the RE200 compared to the CON. Some selected up-regulated DEGs (*CLDN2*, *TLR5*, *MMP1* and *SDC5*) existed in the magnum samples, have been positively related to several functions, including barrier function [47], antimicrobial defense [48], and albumen synthesis and/or secretion [49, 50]. It is also worth noting that the gene expressions of *CACNA1B*, *SLC6A17* and *SERPINB10* were significantly up-regulated in the magnum samples of RE200 group. The gene *CACNA1B* is related to several important biological processes, including protein and lipid metabolism [51]. The solute carriers (SLCs), including SLC1A4, SLC6A17, SLC7A7 and SLC7A11, are membrane transporters that accelerate the transport of precursor molecules for protein synthesis [49]. The SERPIN family can modulate the functions of the oviductal magnum for egg albumen formation [49]. Moreover, proteomic data of albumen suggested that the SERPIN proteins can be incorporated in egg albumen [52]. The increased gene expressions in the oviductal magnum indicated that they actively participated in the synthesis of egg albumen in laying hens fed with RE. According to KEGG enrichment analysis, four pathways were significantly enriched in the RE200 group, which were closely associated with immune response (relaxin signaling pathway) [53], albumen formation and secretion (focal adhesion and ECM-receptor interaction) [50], and behavior (circadian entrainment) [54]. Collectively, RNA-seq data showed that RE improved gene expressions and functional pathways related to immune function and albumen formation in the oviductal magnum, which provided a direct molecular mechanism for improvements in egg albumen of laying hens induced by RE supplementation.

## Conclusion

Here, we provided a finding that 200 mg/kg rosemary extract effectively enhanced egg quality in late-phase laying hens via modulating gut barrier function, gut microbiota, and oviductal function, including upregulating the gene expressions of jejunal *ZO-1* and *Occludin*, enhancing the abundances of cecal Firmicutes, *Eisenbergiella*, *Paraprevotella* and *Papillibacter*, increasing the production of butyrate, and modulating gene expressions and functional pathways related to immunity and egg-white formation in the oviductal magnum. The present study provided a theoretical basis for the application of rosemary extract in late-phase laying hens and identified a new strategy to improve egg quality.

### Supplementary Information

**Additional file 1:** **Table S1.** Ingredients and nutrient composition of the basal diet.

**Additional file 2:** **Table S2.** Primer sequences for quantitative real-time PCR.

**Additional file 3:** **Table S3.** The yield, purity, and RIN value of RNA extracted from oviductal magnum.

**Additional file 4:** **Table S4.** Summary of read features derived from the RNA-seq analysis.

**Additional file 5:** **Table S5.** Top 75 up-regulated DEGs in the oviductal magnum of laying hens.

**Additional file 6:** **Table S6.** Top 75 down-regulated DEGs in the oviductal magnum of laying hens.

## Data Availability

All data produced or analyzed during this study are available from the corresponding author on reasonable request.

## Abbreviations

CAT: Catalase
DEGs: Differentially expressed genes
GSH-Px: Glutathione peroxidase
IgA: Immunoglobulin A
IL-1β: Interleukin-1β
IL-6: Interleukin-6
OUT: Operational taxonomic units
RE: Rosemary extract
ROS: Reactive oxygen species
SCFA: Short-chain fatty acids
SLCs: Solute carriers
SOD: Superoxide dismutase
TC: Total cholesterol
TNF-α: Tumor necrosis factor-α
