# Supplementary material for: Rosemary extract improves egg quality by altering gut barrier function, intestinal microbiota and oviductal gene expressions in late-phase laying hens
Source: J Anim Sci Biotechnol. 2023 Sep 4;14:121. doi: 10.1186/s40104-023-00904-6 (PMC10476401; doi:10.1186/s40104-023-00904-6)
Supplement: Supplementary file 1 — Additional file 1: Table S1. Ingredients and nutrient composition of the basal diet. [file 40104_2023_904_MOESM1_ESM.docx]

**Table S1** Ingredients and nutrient composition of the basal diet (%, as-fed basis)

| **Item** | **Content** |
| --- | --- |
| Ingredients |  |
| Corn | 62.00 |
| Soybean meal | 23.00 |
| Soybean oil | 1.00 |
| Limestone | 8.50 |
| Premix^1^ | 5.50 |
| Nutrient levels^2^ |  |
| Metabolizable energy, MJ/kg | 11.06 |
| Crude protein | 15.57 |
| Lysine | 0.78 |
| Methionine | 0.44 |
| Threonine | 0.58 |
| Tryptophan | 0.21 |
| Calcium | 3.97 |
| Total phosphorus | 0.41 |

^1^ Premix provided the following per kg of the diet: vitamin A, 100,000 IU; vitamin D_3_, 35,000 IU; vitamin E, 480 IU; vitamin K_3_, 35 mg; vitamin B_1_, 22 mg; vitamin B_2_, 48 mg; niacin, 400 mg; pantothenic acid, 170 mg; Fe, 1,000 mg; Cu, 150 mg; Mn, 1,000 mg; Zn, 800 mg; Ca, 120 g; P, 16 g; NaCl, 50 g

^2^ Calculated nutrient levels
