# Supplementary material for: Rosemary extract improves egg quality by altering gut barrier function, intestinal microbiota and oviductal gene expressions in late-phase laying hens
Source: J Anim Sci Biotechnol. 2023 Sep 4;14:121. doi: 10.1186/s40104-023-00904-6 (PMC10476401; doi:10.1186/s40104-023-00904-6)
Supplement: Supplementary file 2 — Additional file 2: Table S2. Primer sequences for quantitative real-time PCR. [file 40104_2023_904_MOESM2_ESM.docx]

**Table S2** Primer sequences for quantitative real-time PCR

| **Gene** | **Primer sequences (5' to 3')** |
| --- | --- |
| *IL-6* | F: CTCCTCGCCAATCTGAAGTC |
|  | R: CCTCACGGTCTTCTCCATAAAC |
| *TNF-α* | F: GGACAGCCTATGCCAACAAG |
|  | R: GCGGTCATAGAACAGCACTAC |
| *IFN-γ* | F: CTTCCTGATGGCGTGAAGA |
|  | R: GAGGATCCACCAGCTTCTGT |
| *ZO-1* | F: ACAGCTCATCACAGCCTCCT |
|  | R: TGAAGGGCTTACAGGAATGG |
| Occludin | F: AGTTCGACACCGACCTGAAG |
|  | R: TCCTGGTATTGAGGGCTGTC |
| Claudin-1 | F: AAGTGCATGGAGGATGACCA |
|  | R: GCCACTCTGTTGCCATACCA |
| *MUC-2* | F: GCCTGCCCAGGAAATCAAG |
|  | R: CGACAAGTTTGCTGGCACAT |
| *CLDN2* | F: CTGCTCACCCTCATTGGA |
|  | R: AACTCACTCTTGGGCTTCTG |
| *MMP1* | F: CTCTGAAAGTCTGGAGCAGT |
|  | R: ACAAGTTGTAGCCTCTTCCA |
| *TLR5* | F: CACTGCTGGAGGATTTGTTCTTG |
|  | R: ACAGACGGAGTATGGTCAAACG |
| *SDC3* | F: CATCAGCAACAGGACACAGT |
|  | R: GGAGCAGGAGCCAAGAAG |
| *CAPN2* | F: GACCTGCTAGATAACGATGG |
|  | R: ATACGAGTTCATGGTTCCAG |
| β-actin | F: GAGAAATTGTGCGTGACATCA |
|  | R: CCTGAACCTCTCATTGCCA |

*IL-6*, interleukin-6; *TNF-α*, tumor necrosis factor-α; *IFN-γ*, interferon-γ; *ZO-1*, zonula occludens-1; *MUC-2*, mucin-2; CLDN2, claudin-2; *MMP1*, matrix metallopeptidase 1; *TLR5*, toll like receptor 5; *SDC3*, syndecan 3; *CAPN2*, calpain 2
