# Supplementary material for: Rosemary extract improves egg quality by altering gut barrier function, intestinal microbiota and oviductal gene expressions in late-phase laying hens
Source: J Anim Sci Biotechnol. 2023 Sep 4;14:121. doi: 10.1186/s40104-023-00904-6 (PMC10476401; doi:10.1186/s40104-023-00904-6)
Supplement: Supplementary file 3 — Additional file 3: Table S3. The yield, purity, and RIN value of RNA extracted from oviductal magnum. [file 40104_2023_904_MOESM3_ESM.docx]

**Table S3** The yield, purity, and RIN value of RNA extracted from oviductal magnum

| **Group** | **Replicate** | **RNA yield, ng/μL** | **OD_260/280_** | **RIN** |
| --- | --- | --- | --- | --- |
| CON | 1 | 2226.50 | 2.06 | 10.00 |
|  | 2 | 1568.10 | 2.10 | 9.70 |
|  | 3 | 1350.90 | 2.10 | 9.70 |
|  | 4 | 2325.10 | 2.05 | 9.60 |
|  | 5 | 2040.00 | 2.11 | 10.00 |
|  | 6 | 1300.80 | 2.13 | 9.60 |
| RE200 | 1 | 1646.80 | 2.11 | 9.90 |
|  | 2 | 2176.40 | 2.08 | 9.60 |
|  | 3 | 2370.20 | 2.05 | 10.00 |
|  | 4 | 2020.50 | 2.08 | 9.70 |
|  | 5 | 1909.60 | 2.09 | 9.70 |
|  | 6 | 2179.10 | 2.05 | 9.90 |

*CON*, control; *RE200*, 200 mg/kg rosemary extract
