# Supplementary material for: Rosemary extract improves egg quality by altering gut barrier function, intestinal microbiota and oviductal gene expressions in late-phase laying hens
Source: J Anim Sci Biotechnol. 2023 Sep 4;14:121. doi: 10.1186/s40104-023-00904-6 (PMC10476401; doi:10.1186/s40104-023-00904-6)
Supplement: Supplementary file 4 — Additional file 4: Table S4. Summary of read features derived from the RNA-seq analysis. [file 40104_2023_904_MOESM4_ESM.docx]

**Table S4** Summary of read features derived from the RNA-seq analysis

| **Group** | **Replicate** | **Raw reads** | **Clean reads** | **Mapped** | **Mapping rate, %** |
| --- | --- | --- | --- | --- | --- |
| CON | 1 | 53984764 | 53698338 | 50431532 | 93.92 |
|  | 2 | 48441520 | 48193862 | 44954309 | 93.28 |
|  | 3 | 50035092 | 49784076 | 48038746 | 96.49 |
|  | 4 | 48398770 | 48058488 | 45660902 | 95.01 |
|  | 5 | 46241648 | 45991444 | 43718979 | 95.06 |
|  | 6 | 53948718 | 53586572 | 51205292 | 95.56 |
| RE200 | 1 | 54738286 | 54386504 | 51438764 | 94.58 |
|  | 2 | 54311822 | 53742910 | 49496198 | 92.10 |
|  | 3 | 65317936 | 64940908 | 61527109 | 94.74 |
|  | 4 | 59950550 | 59596202 | 56502435 | 94.81 |
|  | 5 | 48677208 | 48445702 | 46653093 | 96.30 |
|  | 6 | 56175096 | 55837578 | 53704130 | 96.18 |

*CON*, control; *RE200*, 200 mg/kg rosemary extract
