# Supplementary material for: Rosemary extract improves egg quality by altering gut barrier function, intestinal microbiota and oviductal gene expressions in late-phase laying hens
Source: J Anim Sci Biotechnol. 2023 Sep 4;14:121. doi: 10.1186/s40104-023-00904-6 (PMC10476401; doi:10.1186/s40104-023-00904-6)
Supplement: Supplementary file 5 — Additional file 5: Table S5. Top 75 up-regulated DEGs in the oviductal magnum of laying hens. [file 40104_2023_904_MOESM5_ESM.docx]

**Table S5** Top 75 up-regulated DEGs in the oviductal magnum of laying hens (RE200 vs. CON)

| **Gene ID** | **Gene name** | **Gene description** | **Log_2_(FC)** | ***P*-value** |
| --- | --- | --- | --- | --- |
| ENSGALG00000008456 | CACNA1B | Calcium voltage-gated channel subunit alpha1 B | 5.11 | 0.004 |
| ENSGALG00000000394 | SLC6A17 | Solute carrier family 6 member 17 | 4.76 | 0.048 |
| ENSGALG00000019552 | SERPINB10 | Serpin family B member 2 | 4.58 | 0.002 |
| ENSGALG00000008177 | NOS1 | Nitric oxide synthase 1 | 4.31 | 0.000 |
| ENSGALG00000044618 | ENSGALG00000044618 | Solute carrier family 51 beta subunit | 4.24 | 0.014 |
| ENSGALG00000043139 | SYT7 | Synaptotagmin VII | 4.05 | 0.016 |
| ENSGALG00000000745 | SLC26A9 | Solute carrier family 26 member 9 | 3.99 | 0.045 |
| ENSGALG00000028803 | B3GNT4 | UDP-GlcNAc:betaGal beta-1,3-N-acetylglucosaminyltransferase 4 | 3.77 | 0.025 |
| ENSGALG00000007311 | CLDN2 | Claudin 2 | 3.73 | 0.030 |
| ENSGALG00000016837 | ENSGALG00000016837 | Myosin XVI | 3.71 | 0.007 |
| ENSGALG00000016992 | HTR2A | 5-hydroxytryptamine receptor 2A | 3.70 | 0.012 |
| ENSGALG00000023355 | TMEM154 | Transmembrane protein 154 | 3.64 | 0.003 |
| ENSGALG00000045153 | SHISA3 | Shisa family member 3 | 3.59 | 0.015 |
| ENSGALG00000028415 | ENSGALG00000028415 | Hairy and enhancer of split 6 (Drosophila) | 3.53 | 0.003 |
| ENSGALG00000019061 | MMP1 | Matrix metallopeptidase 1 | 3.47 | 0.013 |
| ENSGALG00000016448 | KCNF1 | Potassium voltage-gated channel modifier subfamily F member 1 | 3.42 | 0.003 |
| ENSGALG00000034392 | ENSGALG00000034392 | - | 3.29 | 0.001 |
| ENSGALG00000010866 | AREG | Amphiregulin | 3.19 | 0.023 |
| ENSGALG00000013740 | SLC20A1 | Solute carrier family 20 member 1 | 3.08 | 0.000 |
| ENSGALG00000001534 | TMEM88B | Transmembrane protein 88B | 3.05 | 0.029 |
| ENSGALG00000001276 | HOXB9 | Homeobox B9 | 2.97 | 0.001 |
| ENSGALG00000049649 | ENSGALG00000049649 | - | 2.96 | 0.017 |
| ENSGALG00000016774 | NPAS2 | Neuronal PAS domain protein 2 | 2.95 | 0.000 |
| ENSGALG00000023772 | HSPB7 | Heat shock protein family B (small) member 7 | 2.94 | 0.000 |
| ENSGALG00000006186 | RAD21L1 | RAD21 cohesin complex component like 1 | 2.86 | 0.006 |
| ENSGALG00000005903 | NRIP3 | Nuclear receptor interacting protein 2 | 2.86 | 0.000 |
| ENSGALG00000009439 | GPR85 | G protein-coupled receptor 85 | 2.83 | 0.018 |
| ENSGALG00000012538 | NR4A2 | Nuclear receptor subfamily 4 group A member 2 | 2.82 | 0.005 |
| ENSGALG00000033696 | RALY | RALY heterogeneous nuclear ribonucleoprotein | 2.78 | 0.000 |
| ENSGALG00000020538 | SLC49A3 | Major facilitator superfamily domain containing 7 | 2.78 | 0.000 |
| ENSGALG00000010812 | RYR2 | Ryanodine receptor 2 | 2.78 | 0.000 |
| ENSGALG00000047554 | GDF7 | Growth differentiation factor 7 | 2.75 | 0.000 |
| ENSGALG00000017099 | RASL11A | Ras-like family 11 member A | 2.73 | 0.035 |
| ENSGALG00000005401 | WNT9A | Wnt family member 9A | 2.71 | 0.000 |
| ENSGALG00000045925 | ENSGALG00000045925 | - | 2.70 | 0.018 |
| ENSGALG00000053524 | ENSGALG00000053524 | - | 2.69 | 0.019 |
| ENSGALG00000013568 | NR4A3 | Nuclear receptor subfamily 4 group A member 3 | 2.69 | 0.000 |
| ENSGALG00000004969 | TIMP4 | TIMP metallopeptidase inhibitor 4 | 2.69 | 0.018 |
| ENSGALG00000039634 | KRT80 | Keratin 80 | 2.68 | 0.044 |
| ENSGALG00000051258 | ENSGALG00000051258 | - | 2.68 | 0.007 |
| ENSGALG00000029072 | NTN3 | Netrin 1 | 2.62 | 0.014 |
| ENSGALG00000006560 | SEMA3A | Semaphorin 3A | 2.61 | 0.014 |
| ENSGALG00000009422 | RGS10 | Regulator of G-protein signaling 10 | 2.58 | 0.000 |
| ENSGALG00000016265 | LANCL3 | LanC like 3 | 2.54 | 0.024 |
| ENSGALG00000040079 | ZFPM2 | Zinc finger protein, FOG family member 2 | 2.52 | 0.049 |
| ENSGALG00000031255 | FGF1 | Fibroblast growth factor 1 | 2.52 | 0.035 |
| ENSGALG00000007174 | TNFSF15 | Tumor necrosis factor superfamily member 15 | 2.51 | 0.000 |
| ENSGALG00000011835 | FGF2 | Fibroblast growth factor 2 | 2.45 | 0.001 |
| ENSGALG00000004400 | ENSGALG00000004400 | Sperm antigen with calponin homology and coiled-coil domains 1 | 2.44 | 0.005 |
| ENSGALG00000005739 | SCD | Stearoyl-CoA desaturase | 2.43 | 0.000 |
| ENSGALG00000001768 | TENM2 | Teneurin transmembrane protein 2 | 2.40 | 0.005 |
| ENSGALG00000007503 | KMO | Kynurenine 3-monooxygenase | 2.40 | 0.020 |
| ENSGALG00000005460 | SLCO4A1 | Solute carrier organic anion transporter family member 4A1 | 2.36 | 0.016 |
| ENSGALG00000001203 | ENSGALG00000001203 | Transmembrane protein 45B | 2.35 | 0.001 |
| ENSGALG00000009095 | LHCGR | Luteinizing hormone/choriogonadotropin receptor | 2.34 | 0.045 |
| ENSGALG00000002563 | TPM4 | Tropomyosin 4 | 2.32 | 0.017 |
| ENSGALG00000015494 | HTR1F | 5-hydroxytryptamine receptor 1F | 2.31 | 0.026 |
| ENSGALG00000009392 | TLR5 | Toll like receptor 5 | 2.29 | 0.050 |
| ENSGALG00000013297 | BBOX1 | Gamma-butyrobetaine hydroxylase 1 | 2.29 | 0.005 |
| ENSGALG00000038133 | SMIM3 | Small integral membrane protein 3 | 2.29 | 0.000 |
| ENSGALG00000016797 | FHL2 | Four and a half LIM domains 2 | 2.28 | 0.001 |
| ENSGALG00000011970 | CHADL | Chondroadherin-like | 2.28 | 0.002 |
| ENSGALG00000001573 | P2RX1 | Purinergic receptor P2X 1 | 2.28 | 0.040 |
| ENSGALG00000023818 | HSPB9 | Heat shock protein family B (small) member 9 | 2.27 | 0.000 |
| ENSGALG00000039978 | SLC4A1 | Solute carrier family 4 member 1 | 2.24 | 0.009 |
| ENSGALG00000038265 | CREB5 | cAMP responsive element binding protein 5 | 2.23 | 0.028 |
| ENSGALG00000014913 | ROS1 | ROS proto-oncogene 1, receptor tyrosine kinase | 2.22 | 0.000 |
| ENSGALG00000039092 | STAC | SH3 and cysteine rich domain | 2.21 | 0.000 |
| ENSGALG00000027514 | SCEL | Sciellin | 2.21 | 0.008 |
| ENSGALG00000048624 | SGPP2 | Sphingosine-1-phosphate phosphatase 2 | 2.19 | 0.012 |
| ENSGALG00000047043 | ENSGALG00000047043 | Dynein axonemal heavy chain 17 | 2.18 | 0.016 |
| ENSGALG00000005215 | CACNA1H | Calcium voltage-gated channel subunit alpha1 H | 2.17 | 0.000 |
| ENSGALG00000004758 | KCNB1 | Potassium voltage-gated channel subfamily B member 1 | 2.16 | 0.024 |
| ENSGALG00000048672 | ENSGALG00000048672 | Uncharacterized LOC112530909 | 2.15 | 0.007 |
| ENSGALG00000010825 | AGR2 | Anterior gradient 2, protein disulphide isomerase family member | 2.15 | 0.007 |

*DEGs*, differentially expressed genes; *FC*, fold change; *CON*, control; *RE200*, 200 mg/kg rosemary extract. *n* = 6
