# Supplementary material for: Rosemary extract improves egg quality by altering gut barrier function, intestinal microbiota and oviductal gene expressions in late-phase laying hens
Source: J Anim Sci Biotechnol. 2023 Sep 4;14:121. doi: 10.1186/s40104-023-00904-6 (PMC10476401; doi:10.1186/s40104-023-00904-6)
Supplement: Supplementary file 6 — Additional file 6: Table S6. Top 75 down-regulated DEGs in the oviductal magnum of laying hens. [file 40104_2023_904_MOESM6_ESM.docx]

**Table S6** Top 75 down-regulated DEGs in the oviductal magnum of laying hens (RE200 vs. CON)

| **Gene ID** | **Gene name** | **Description** | **Log_2_(FC)** | ***P*-value** |
| --- | --- | --- | --- | --- |
| ENSGALG00000047578 | SELENOP1 | Selenoprotein P1 | -6.38 | 0.018 |
| ENSGALG00000006989 | ENSGALG00000006989 | Heparan sulfate glucosamine 3-O-sulfotransferase 1-like | -6.23 | 0.000 |
| ENSGALG00000053508 | ENSGALG00000053508 | - | -5.71 | 0.000 |
| ENSGALG00000050628 | ENSGALG00000050628 | - | -5.59 | 0.000 |
| ENSGALG00000011624 | PNLDC1 | PARN like, ribonuclease domain containing 1 | -5.16 | 0.005 |
| ENSGALG00000010497 | SLC29A4 | Solute carrier family 29 member 4 | -5.15 | 0.000 |
| ENSGALG00000006693 | ENSGALG00000006693 | BPI fold-containing family B member 4-like | -4.99 | 0.000 |
| ENSGALG00000049890 | ENSGALG00000049890 | - | -4.98 | 0.005 |
| ENSGALG00000051683 | ENSGALG00000051683 | - | -4.90 | 0.032 |
| ENSGALG00000017172 | VMO1 | Vitelline membrane outer layer 1 homolog | -4.77 | 0.001 |
| ENSGALG00000054601 | ENSGALG00000054601 | - | -4.62 | 0.019 |
| ENSGALG00000035539 | HOXC4 | Homeobox C4 | -4.61 | 0.018 |
| ENSGALG00000053641 | ENSGALG00000053641 | Circadian associated repressor of transcription | -4.58 | 0.001 |
| ENSGALG00000047394 | ENSGALG00000047394 | - | -4.48 | 0.012 |
| ENSGALG00000050654 | ENSGALG00000050654 | - | -4.46 | 0.000 |
| ENSGALG00000038740 | AMY2A | Amylase, alpha 2A (pancreatic) | -4.34 | 0.002 |
| ENSGALG00000039826 | CNGA3 | Cyclic nucleotide gated channel alpha 3 | -4.18 | 0.001 |
| ENSGALG00000037841 | GRM7 | Glutamate metabotropic receptor 7 | -4.06 | 0.000 |
| ENSGALG00000054910 | ENSGALG00000054910 | - | -3.95 | 0.018 |
| ENSGALG00000020982 | ENSGALG00000020982 | Bactericidal/permeability-increasing protein-like 3 | -3.91 | 0.000 |
| ENSGALG00000006755 | USP2 | Ubiquitin specific peptidase 2 | -3.89 | 0.000 |
| ENSGALG00000050806 | ENSGALG00000050806 | Uncharacterized LOC423629 | -3.89 | 0.001 |
| ENSGALG00000011369 | LHX8 | LIM homeobox 8 | -3.87 | 0.000 |
| ENSGALG00000040954 | GPR37 | G protein-coupled receptor 37 | -3.73 | 0.000 |
| ENSGALG00000047988 | ENSGALG00000047988 | - | -3.72 | 0.006 |
| ENSGALG00000037200 | ENSGALG00000037200 | MACRO domain containing 2 | -3.61 | 0.000 |
| ENSGALG00000008552 | MAL | Mal, T cell differentiation protein | -3.60 | 0.007 |
| ENSGALG00000030855 | ENSGALG00000030855 | - | -3.54 | 0.049 |
| ENSGALG00000034684 | ENSGALG00000034684 | - | -3.54 | 0.014 |
| ENSGALG00000054252 | TM4SF1 | Transmembrane 4 L six family member 1 | -3.54 | 0.010 |
| ENSGALG00000052583 | ENSGALG00000052583 | - | -3.52 | 0.000 |
| ENSGALG00000053459 | ENSGALG00000053459 | - | -3.52 | 0.009 |
| ENSGALG00000007269 | GABRA3 | Gamma-aminobutyric acid type A receptor alpha3 subunit | -3.47 | 0.000 |
| ENSGALG00000012647 | RFX4 | Regulatory factor X4 | -3.40 | 0.000 |
| ENSGALG00000051075 | ENSGALG00000051075 | - | -3.29 | 0.013 |
| ENSGALG00000034204 | ENSGALG00000034204 | Keratin, type I cytoskeletal 17-like | -3.22 | 0.016 |
| ENSGALG00000030773 | CP | Ceruloplasmin | -3.20 | 0.000 |
| ENSGALG00000052297 | ENSGALG00000052297 | - | -3.15 | 0.004 |
| ENSGALG00000032836 | ENSGALG00000032836 | Monoamine oxidase B | -3.08 | 0.007 |
| ENSGALG00000039772 | RGS7 | Regulator of G-protein signaling 7 | -3.07 | 0.018 |
| ENSGALG00000053789 | ENSGALG00000053789 | - | -3.07 | 0.012 |
| ENSGALG00000031410 | ENPEP | Glutamyl aminopeptidase | -3.06 | 0.001 |
| ENSGALG00000043734 | LIPG | Lipase G, endothelial type | -3.02 | 0.000 |
| ENSGALG00000028376 | FGF19 | Fibroblast growth factor 19 | -2.97 | 0.011 |
| ENSGALG00000043027 | HEPHL1 | Hephaestin like 1 | -2.96 | 0.046 |
| ENSGALG00000016495 | ENSGALG00000016495 | - | -2.89 | 0.001 |
| ENSGALG00000035784 | ENSGALG00000035784 | - | -2.89 | 0.004 |
| ENSGALG00000000893 | TAT | Tyrosine aminotransferase | -2.87 | 0.018 |
| ENSGALG00000012197 | ACMSD | Aminocarboxymuconate semialdehyde decarboxylase | -2.85 | 0.048 |
| ENSGALG00000048023 | ENSGALG00000048023 | - | -2.84 | 0.014 |
| ENSGALG00000005217 | MOGAT1 | Monoacylglycerol O-acyltransferase 1 | -2.81 | 0.000 |
| ENSGALG00000038432 | ENSGALG00000048023 | - | -2.80 | 0.037 |
| ENSGALG00000002742 | TMEM132B | Transmembrane protein 132B | -2.78 | 0.000 |
| ENSGALG00000008599 | ENSGALG00000008599 | Carbohydrate sulfotransferase 9-like | -2.76 | 0.004 |
| ENSGALG00000006666 | BPIFB2 | BPI fold containing family B member 2 | -2.70 | 0.001 |
| ENSGALG00000031430 | ENSGALG00000031430 | Selectin E | -2.68 | 0.000 |
| ENSGALG00000000678 | CITED4 | Cbp/p300 interacting transactivator with Glu/Asp rich carboxy-terminal domain 4 | -2.66 | 0.000 |
| ENSGALG00000025896 | PLPPR4 | Phospholipid phosphatase related 4 | -2.64 | 0.024 |
| ENSGALG00000031906 | FAM83C | Family with sequence similarity 83 member C | -2.60 | 0.000 |
| ENSGALG00000040949 | ENSGALG00000040949 | - | -2.58 | 0.002 |
| ENSGALG00000033012 | SPRY4 | Sprouty RTK signaling antagonist 4 | -2.58 | 0.011 |
| ENSGALG00000021340 | CA9 | Carbonic anhydrase 9 | -2.57 | 0.008 |
| ENSGALG00000009920 | COCH | Cochlin | -2.54 | 0.004 |
| ENSGALG00000002466 | SLC2A5 | Solute carrier family 2 member 5 | -2.53 | 0.037 |
| ENSGALG00000050720 | ENSGALG00000050720 | - | -2.50 | 0.020 |
| ENSGALG00000008701 | XDH | Xanthine dehydrogenase | -2.44 | 0.011 |
| ENSGALG00000045037 | CYP2W1 | Cytochrome P450, family 2, subfamily W, polypeptide 2 | -2.40 | 0.000 |
| ENSGALG00000036447 | ENSGALG00000036447 | - | -2.38 | 0.015 |
| ENSGALG00000015263 | ENSGALG00000015263 | Transmembrane protein 30C, pseudogene | -2.36 | 0.011 |
| ENSGALG00000001933 | CORO2A | Coronin 2A | -2.36 | 0.010 |
| ENSGALG00000028975 | ENSGALG00000028975 | FERM and PDZ domain containing 3 | -2.34 | 0.026 |
| ENSGALG00000000295 | ENSGALG00000000295 | Immunoglobulin-like and fibronectin type III domain containing 1 | -2.33 | 0.000 |
| ENSGALG00000013722 | SEMA3B | Semaphorin 3B | -2.33 | 0.000 |
| ENSGALG00000011200 | THBS2 | Thrombospondin 2 | -2.32 | 0.000 |
| ENSGALG00000053559 | ENSGALG00000053559 | - | -2.32 | 0.000 |

*DEGs*, differentially expressed genes; *FC*, fold change; *CON*, control; *RE200*, 200 mg/kg rosemary extract. *n* = 6
